# Ophthalmic Drug Delivery in Glaucoma—A Review

**DOI:** 10.3390/pharmaceutics4010243

**Published:** 2012-03-21

**Authors:** Ingrida Januleviciene, Lina Siaudvytyte, Ruta Barsauskaite

**Affiliations:** Eye Clinic of Lithuanian University of Health Sciences, Kaunas 50009, Lithuania

**Keywords:** glaucoma, pharmacokinetics, drug penetration, topical application

## Abstract

Glaucoma is a progressive optic neuropathy and medical therapy is the initial option for the treatment of this potentially blinding condition. Topical instillation of eye drops from the bottle is the most common glaucoma drug delivery form. Due to limited permeability of anterior ocular surface, natural clearance and drainage, eye drops contain large amounts of inactive ingredients. Effective penetration enhancers are known as irritants causing ocular discomfort. Although drug efficacy is determined by active ingredients, inactive agents can affect tolerance and can result in conjunctival irritation and hyperemia and influence patients’ adherence and quality of life.

## 1. Introduction

Glaucoma is a progressive optic neuropathy and medical therapy is the initial option for the treatment of this potentially blinding condition. Topical instillation of eye drops from the bottle is the common ophthalmic drug delivery form. Eye drops usually penetrate via corneal or scleral route, although some conjunctival contribution is noted [1,2]. The administration of pharmacological compounds from drip bottles sometimes can be problematic for a variety of reasons. First, the anterior ocular surface has limited permeability and is continuously washed by tears. The lacrimal apparatus and nasolacrimal duct drains tears and other substances from the eye to the nasal cavity. Due to limited permeability of anterior ocular surface, natural clearance and drainage, eye drops contain large amounts of inactive ingredients. Effective penetration enhancers are known as irritants causing ocular discomfort [3]. Other disadvantages of topically used eye drops include problematic treatment schedules and difficulty in application of eye drops. Various adverse effects associated with topical medication may have a negative effect on patient adherence to medical treatment, doctor-patient relationship and patient quality of life [4]. However, topical drugs have clear topical administration advantages and constitute a more convenient way of administration as well as avoiding hepatic first-pass metabolism [2]. Current implantable drug delivery devices addressing patient non-compliance and fluctuations of intraocular pressure (IOP) issues, however, also have a clear limitation—it is not possible to change, increase or decrease, or stop drug delivery once it is introduced into the eye. For chronic conditions such as glaucoma, it would be optimal to regulate drug delivery depending on the therapeutic response and progression of the disease. Another difficulty with the implantable drug delivery system is that the surgical procedure for implanting is invasive and requires skillful vitreoretinal surgeon. Clinical testing is provided for drug-eluting punctal plugs investigated as sustained-release drug delivery systems for some glaucoma medication. The studies have not yet been published, but initial data from one of the trials indicates that the device did not significantly lower IOP [5].

## 2. Physiological Aspects of Topical Drug Delivery to the Eye

The drug concentration at the receptor site is a critical determinant of rate of onset, intensity and duration of a pharmacological effect. The drug effect depends on its activity, affinity for a receptor or enzyme and ability to reach the site of action in sufficient concentration. Drug pharmacokinetics investigates drug absorption, distribution and elimination within the body [1,6]. Topically administered drugs on the delivery to the site of action face medias with different vascularization (from highly vascularized inner retina to avascular lens or cornea), as well as multiform consistency tissues, from liquid aqueous humor to solid lens, thus determining different drug diffusion [2]. After topical administration through absorption process, a drug enters the aqueous humor. Absorption is influenced by drug solubility in tears and ocular surface permeability. Conjunctival and scleral tissues have similar permeability to hydrophilic drugs, while cornea is 15–25 times less permeable [7]. Bioavailability in ophthalmology refers to the amount of drug entering the aqueous humor. The drug is further transferred and distributed within intraocular tissues—conjunctiva, cornea, lens, iris, ciliary body, choroid, vitreous body, retina and optic nerve. Several factors might influence availability of topical ophthalmic medication: flush by tear film, limited capacity of conjunctival cul-de-sac, dilution by tears and aqueous humor, drainage into the nasolacrimal duct, binding to melatonin or proteins, metabolism within ocular tissues. All ocular tissues are able to accumulate drugs. Large conjunctival surface and nasal mucosa allows a portion of topical drug that is not absorbed into the eye to enter the systemic circulation. Elimination from the eye occurs usually during aqueous humor turnover or passage across blood-ocular barrier.

Various approaches are used to increase bioavailability of eye drops by increasing corneal penetration or drops viscosity. Ocular absorption is increased by adding cyclodextrins, solid inserts and colloidal systems to ophthalmic drugs. Higher viscosity drops are constituted of high molecular weight molecules hardly crossing biological membranes. Having a longer wash-out from the tear film viscous drops stay longer on periocular surface and increase drug delivery to the deeper ocular structures [2]. On the other hand, high viscosity interferes with eyelid movements, vision [8] and patient comfort.

Economic situation obligates seeking for cheaper and generally conventional treatment options. Still some doubts exist if generics are exactly as effective and tolerable as branded drugs. Even having the same active ingredient, bioequivalence, however, can not be guarantied. Different size of drug particulates and pH can change its pharmacokinetics and distribution in tissues. Moreover different inactive ingredients and preservatives can determine different penetration, absorption and availability of the active agent at the site of action [9]. Ocular surface sensitivity to inactive ingredients and preservatives in ophthalmic preparations, which are known to vary between generics and branded agents, may considerably alter distribution of drug within tissues and tolerability. Slight alteration in the IOP-lowering efficacy of anti-glaucoma drugs can have a deleterious effect on the eyes in the long-term, as it is well-known that even slight increase in IOP can aggravate progression of glaucomatous visual field loss.

## 3. Importance of Tear Film

The tear film is essential for maintaining the health of the cornea and conjunctiva. Since the tear film is the first and most powerful refracting surface of the eye, irregularities in the tear film thickness can cause optical aberrations in the eye. To maintain a healthy ocular surface the quantity of tears is important, but also proper chemical composition in order to nourish and protect ocular surface cells. The tear film consists of: (1) lipid component containing wax esters, sterol esters, fatty acids and fatty alcohols; (2) mucous component comprised of mucins that are constituted largely of sugars; (3) aqueous component, which constitutes the bulk of the tear film, composed of 98% water but also salts, mucins, and proteins including hyaluronan, lysozyme, lactoferrin, lipocalin, and secretory immunoglobulins [10]. Disruption of the homeostasis of the tear film results in ocular surface inflammation, which may lead to cell damage. Abnormalities of any tear component can result in tear film instability and hyperosmolarity [11,12,13]. 

The pH of healthy tears is reported to range from 7.3 to 7.7, it is influenced by dissolved substances, especially by the bicarbonate–carbon dioxide buffer system. Tear pH is lowest upon wakening due to acid byproducts associated with prolonged eyelid closure. When the eyelids are open, pH increases rapidly due to carbon dioxide loss [14]. It is known that eye drops within pH 6–9 range do not cause discomfort, while drops with pH outside these levels increase production of tear fluid due to irritation and decrease its bioavailability by overflowing drug [2].

Osmolarity is the measure of solute concentration, defined as the number of osmoles (Osm) of solute per liter (L) of solution (osmol/L or Osm/L) [15]. As a measure of tear film chemistry, osmolarity can be useful for evaluating the quality of patients’ tears. In general terms, osmolarity describes the quantity of solutes in a solution; in tears, it specifically refers to the concentration of small proteins and electrolytes, including sodium, potassium, and chloride. Although measuring osmolarity does not reveal the exact chemical composition of tears, it quantifies how concentrated they are, and research has shown that knowledge of tear film osmolarity can be clinically valuable for assessing dry eye disease. Tear film osmolarity could be either too low or too high.

According to the generally accepted concept, the tears are isosmotic with a 1.4 per cent sodium chloride solution, and the recommendation for adjusting the osmotic concentration of collyriums to this presumed tonicity has found worldwide acceptance. Human tear film—305–310 mOsm/L. 

Hyperosmolarity causes ocular surface cell damage, which can be visualized by ocular surface staining. This damage occurs because ocular surface cell membranes are permeable; when they are exposed to hyperosmotic tears, water flows out of the cells in an attempt to balance the osmolarity of the intracellular fluid with the osmolarity of the surrounding tears. When this happens, ocular surface cells can become dehydrated, which damages cell membranes and changes the way proteins protect the ocular surface.

A hypoosmolarity of 150 mOsm/L is subjectively well accepted by patients, but 75 mOsm/L produces irritation of the eye. The absolute hypoosmolarity (0 mOsm/L) is distillated water, which causes itching and swelling of the epithelium.

## 4. Innervation of the Ocular Surface

The exposed surface of the eye is richly innervated by sensory nerve fibers originated at trigeminal ganglion neurons. They reach the cornea and bulbar conjunctiva as thin myelinated or unmyelinated nerve fibers lacking of morphological terminal specialization. However, electrophysiological studies have shown that sensory neurons innervating the eye are functionally heterogeneous. Based upon their response to specific stimuli, different functional types of sensory nerve fibers have been identified in the cornea and bulbar conjunctiva. Mechanonociceptor fibers (~20% of the total) react only to mechanical forces; polymodal nociceptor fibers (~70%) respond to mechanical forces but also to heat, exogenous chemical irritants and endogenous inflammatory mediators; cold-sensitive fibers (~10–15%) display an ongoing impulse activity at basal corneal temperatures and increase markedly their firing frequency with moderate cooling. During inflammation, surgical injury, dryness of the ocular surface activity of ocular sensory nerve fibers changes markedly as the result of short-term changes in ion channel expression secondary to local release of inflammatory agents and growth factors, and of long-lasting modifications in gene expression. This leads to the development of spontaneous activity and of abnormal responsiveness to natural stimuli. In addition to their role in the production of conscious innocuous and noxious sensations referred to the eye surface, sensory fibers appear to play a role in the maintenance of the ocular surface homeostasis, including basal and reflex modulation of tearing and trophic maintenance of corneal and conjunctival tissues [16].

## 5. Effect of Topical Medication on Ocular Surface

Topical glaucoma medications have been associated with ocular surface disease as after instillation drops interact with ocular surface tissues. This interaction can involve the active agents themselves or the preservatives used to keep the bottles sterile and/or to stabilize the active agents in solution. Most preservatives act like detergents and might also influence corneal permeability of topical drugs by causing epithelial separation [17]. The most popular preservatives are the cationic surfactants including the widely used benzalkonium chloride (BAK). As a surfactant, BAK can increase solubility of drugs that are hydrophilic and exert their bactericidal effect by emulsification of bacterial cell walls. Ocular damage from these agents is most likely due to emulsification of the cell membrane lipids [18]. BAK has been shown to be toxic to conjuctival [19] and corneal endothelial cells [20]. It has also been shown to cause opacification increased hydration and corneal thickness [21] and also cause irritation and redness of the eye. BAK being cationic detergent causes epithelial toxicity and is also responsible for a shortening of the tear film break-up time, disruption of surface cell layer and slowing down of the epithelial healing process. Studies have shown that other preservatives had similar effects on ocular surface [22]. Overall, it seems that preservatives damage corneal epithelium but they enhance the permeability of the cornea at the same time. Higher drug penetration is usually associated with a better pharmacological effect. However, the systemic absorption of the drug via the conjunctiva or the nasal mucous layers also enhances [23]. This effect is often problematic for drugs with potent systemic activity, like timolol. It could be argued for the cautious use of these compounds. Nowadays single dose containers are available that do not contain preservatives. It could increase patient’s compliance for those who have sensitive or dry eye but should be considered for older or patients with movement restriction in arms, hands or fingers. One of the ways to increase corneal penetrations is by increasing the lipophilicity of the drug. Latanoprost (Xalatan), travoprost (Travatan) are examples of prodrugs developed for this purpose. The ester group in these compounds increases their lipophilicity and enhances corneal permeability. These prodrugs are then converted into the active drugs, the acidic forms, by the esterase enzymes in the cornea. Prodrugs allow increase penetration into the anterior chamber and may reduce local and systemic side effects by decreasing the concentration of drug required [24].

Ocular surface disease becomes increasingly more common with age and glaucoma is also more common in older age. Elderly patients on long-term glaucoma treatment with multiple topical medications ultimately have an increased risk ocular surface disease that might contribute to poor patient compliance and disease progression [25]. It is also important to note that with age there is an activation of glia within the optic nerve head, an increase in extracellular matrix a decrease in retinal ganglion cells, leading to accelerated progression of the glaucomatous process and more aggressive treatment is required.

## 6. Mechanism of Action of Topical Hypotensive Medications

For the treatment of glaucoma IOP can be lowered by three basic mechanisms: suppression of aqueous humor formation, increase of trabecular outflow and increase of uveal outflow. To influence the conventional outflow pathway, drugs must be delivered to the trabecular meshwork and the longitudinal portion of the ciliary muscle and possibly to the episcleral vessels and myofibroblast of the scleral spur. To influence uveoscleral outflow drugs must be get to the interstitial tissue of the ciliary muscle. To affect aqueous secretion drugs must be targeted to the ciliary processes, which is the chief target of the beta-blockers.

Beta-adrenergic receptors (β1 and β2) are widely distributed in the eye. They are found at the ocular surface, in the ocular vessels, trabecular meshwork, lens epithelium, ciliary body and retina. Β2-receptors predominate in the ocular tissues, including the ciliary processes, where they represent 75–90% or more of the β receptors [26]. Beta blockers are competitive antagonist of the β-adrenergic receptors. They inhibit the activation of these receptors in the ciliary processes by blocking the binding of endogenous adrenergic neurotransmitters. By this blockade cyclic AMP level is decreased and consequently aqueous humor production is suppressed [27]. 

Carbon anhydrase in the eye is most abundant in the ciliary body, mainly type II and IV, but can be found in other ocular tissues as well. Carbonic anhydrase inhibitors (CAI) inhibit the carbonic anhydrase in the ciliary epithelium and reduce the production of bicarbonate ion, which is critical component for active ion transport in aqueous formation. A reduction in bicarbonate limits sodium and fluid transport across the ciliary epithelium and decreases aqueous humor production [27]. 

The primary mechanism by which most prostaglandins (PGs) reduce IOP is by increasing outflow, especially through the uveoscleral outflow pathway, possibly by relaxation of the ciliary muscle and lysis of the extracellular connective tissue matrix [28,29] rather than by reducing aqueous humor production [30]. PGs specifically bind to PG receptors present in almost all ocular tissues.

## 7. Ocular Irritation and Blood Flow

Stimulation of the sphenopalatine ganglion complex, a known phenomenon during ocular irritation can cause an increase in cerebral blood flow and since the sphenopalatine ganglion is a parasympathetic ganglion that sends post-ganglionic fibers to the lacrimal gland, it was suggested that stimulation of the fibers that form the efferent limb of the tear secretion reflex induced this observed rise in ocular blood flow [31]. It remains to be evaluated whether irritation besides subjective discomfort might be useful and have positive effect on ocular circulation. 

Ocular blood flow can be divided into choroidal, retrobulbar, and retinal blood flow. With age, changes in the vasculature as thickening of arteriolar basement membrane, decreased elastin, might cause decreased blood flow, increased resistance to flow and decreased nitric oxide activity. Choroidal blood vessels show a decreased density, lumen diameter and blood volume with age. Combined with an increase in scleral rigidity and systemic blood pressure, this leads to a decrease in ocular blood supply. The retrobulbar circulation undergoes a decrease in flow velocity and increase in resistivity. Retinal blood flow shows a similar decrease in volume and velocity, leading to a decrease in optic nerve head circulation [32]. 

It remains to be seen what the effect of ocular and systemic medications is on ocular blood flow. Most of the topical drug in a conventional-sized drop is absorbed into the blood system across the conjunctiva or in the naso-lacrimal duct or digestive system, and thence it can penetrate the ocular tissues of both eyes. The penetration is controlled by the blood retinal barrier [33]. The systemic route of penetration can provide a major portion of the very small amount of drug found in the vitreous or retina after topical administration and significant contralateral effects have also been reported in humans. When drug molecules enter the vitreous, most likely at its anterior zone, they can progress further to the fundus by diffusion through the gel when it is formed or by convection when it is liquefied. The movement can be visualized by fluorescent tracers or by MRI [34]. 

Most ophthalmic drops are strongly bound to the uveal and retinal tissue. This limits the amount of free drug available to act on the vascular receptors in the early stages of drug penetration into the posterior segment. The binding of a drug should correspond to extending the volume of the vitreous, so that the concentration of the unbound pharmaceutically active form in it will be reduced during the entry stages of administration and prolonged when the treatment is interrupted or terminated. On the other hand drug concentration in the vitreous is not necessarily an indication of action on the retina: it may represent the absence of vasomotive receptors in the retinal tissues or lack of access to them [35]. 

## 8. Conclusions

Glaucoma is a vision threatening disease requiring life-long treatment and patient compliance. Topical IOP lowering has been the golden standard in glaucoma therapy for decades. Having clear advantages of easy dosage and application, together with minimum systemic absorption, but its main disadvantages are poor patient adherence and persistence control. It is still to be investigated whether topical glaucoma medication might improve ocular hemodynamics and visual function which is of crucial importance in the management of glaucoma. 
